# Assessment of Chromium Contamination in Aquatic Environments near Tannery Industries: A Portuguese Case Study

**DOI:** 10.3390/toxics13121068

**Published:** 2025-12-11

**Authors:** Liliana J. G. Silva, Maria J. G. Casimiro, Angelina Pena, Maria J. Campos, André M. P. T. Pereira

**Affiliations:** LAQV, Requimte, Laboratory of Bromatology and Pharmacognosy, Faculty of Pharmacy, University of Coimbra, Polo III, Azinhaga de Stª Comba, 3000-548 Coimbra, Portugal

**Keywords:** tanning industry, heavy metal, chromium, graphite furnace atomic absorption spectrometry, risk assessment, river water

## Abstract

Environmental contamination from industrial activities remains a significant concern, with tanneries being major contributors of chromium (Cr) to aquatic systems. Cr, a heavy metal with multiple oxidation states, varies in toxicity and poses risks to both ecosystems and human health. In Portugal, the Alcanena region is particularly affected, hosting around 60 tanning industries. This study assessed total Cr levels in water from the Alviela River and Carvalho Stream, with particular focus on the impact of a local wastewater treatment plant (WWTP) that processes tannery effluents. Water samples were collected upstream and downstream of the WWTP discharge point. Analytical techniques included graphite furnace atomic absorption spectrometry and inductively coupled plasma optical emission spectroscopy, with a detection limit of 0.33 µg L^−1^. The highest Cr concentration (560 µg L^−1^) was found in the Carvalho Stream, downstream of the WWTP, confirming its contribution to local contamination. In the Alviela River, Cr concentrations ranged from 8 to 50 µg L^−1^ downstream of the WWTP, exceeding the predicted no-effect concentration for aquatic organisms and the safety limit for human consumption (25 µg L^−1^). These findings highlight, for the first time, the ongoing environmental impact of tannery effluents in this region and emphasize the urgent need for improved monitoring and pollution control measures.

## 1. Introduction

Within the One Health concept, humans, wildlife, and ecosystems are increasingly exposed to chemical, physical, and biological stressors, from both natural and anthropogenic sources, whose toxic effects—amplified by climate change—make heavy metals such as chromium (Cr) a significant concern [1].

Cr is the thirteenth most common element in the Earth’s crust [2]. It is a metal with various oxidation states, each with different toxicities, with Cr (III) and Cr (VI) being the most frequent; however, they can be interconverted depending on the environmental conditions. Regarding its toxicity, Cr (III) and Cr (VI) have very distinct toxicities, with the hexavalent form being 500 times more toxic than the trivalent form and classified as carcinogenic to humans (Group 1 by IARC). In aquatic environments, its toxicity affects algae, invertebrates, and vertebrates, causing diverse effects such as nervous system impacts and cytotoxicity in fish, photosynthesis inhibition in algae, and impacts on mollusks. In humans, Cr exposure has been linked to various conditions, including stomach, lung, kidney, and urinary cancers, as well as dermatological, hematological, immune, genitourinary, and gastrointestinal disorders. Cr may also act as an endocrine disruptor, affecting pregnancy outcomes [3,4,5].

Water contamination by Cr has received growing attention in today’s global environmental context due to its extensive use in chemical industries—particularly in the tanning sector, which is the largest consumer of Cr in Europe [6,7,8,9]. The European tanning industry has a turnover of EUR 48 billion and employs 435,000 people. Worldwide, 85% of tanning companies use Cr salts [10]. Human exposure to this metal occurs primarily through water, largely as a result of tannery effluents discharged into surface waters, which subsequently contaminate soils, food sources, and drinking water [11,12]. Although Cr naturally occurs worldwide in surface waters at concentrations of 0.5–2 μg L^−1^, significantly higher levels are reported in polluted regions, especially in areas surrounding tannery industries [13].

Regarding legislation, most European countries have a Cr limit in wastewater of 500 μg L^−1^, although its range varies between 100 and 3000 μg L^−1^ in Germany and Belgium, respectively. Portugal has adopted a value of 2000 μg L^−1^ [5,11,13]. In surface waters, various countries have established environmental quality standards (EQS), ranging between 2 μg L^−1^ (UK) and 16 μg L^−1^ (USA) for acute toxicity and between 1 μg L^−1^ (Canada) and 11 μg L^−1^ (USA) for chronic toxicity [13,14]. Due to its toxicity, the World Health Organization suggests a maximum concentration of 50 μg L^−1^ in drinking water, while in Portugal and most European countries, the limit is even more restrictive, at 25 μg L^−1^ [11,15,16]. Additionally, its toxicity has prompted the European Union to propose restrictions on the use of Cr(VI) [17].

Several analytical methodologies are available for the detection of Cr in water, each offering different levels of sensitivity and suitability. Among spectroscopic techniques, graphite furnace atomic absorption spectrometry (GF-AAS) is commonly used for chromium quantification in water due to its high sensitivity and ability to detect trace concentrations [18,19]. More advanced methods, such as inductively coupled plasma techniques, particularly coupled optical emission spectrometry (ICP-OES) detection or with mass spectrometry detection (ICP-MS), usually provide even lower detection limits and multi-element capability [18,20].

Chromium concentrations in surface waters located near tannery industries show substantial variability across different regions, reflecting differences in industrial practices, wastewater treatment efficiency, and environmental regulation (Table S1, Supporting Information) [21,22,23,24,25]. The lowest concentrations are observed in Brazilian rivers, where values between 0.65 and 8.6 µg L^−1^ suggest relatively limited contamination [22]. Polish rivers also present modest levels, ranging from 8 to 19 µg L^−1^ [25]. In contrast, much higher chromium concentrations are found in the Sebou River in Morocco (1400 µg L^−1^) and in Algeria’s Mouttas River (1170–1463 µg L^−1^), both areas historically influenced by tanning and leather processing activities [23,24]. The most extreme value appears in India’s Hazaribagh region, with concentrations reaching 5190 µg L^−1^, consistent with its intense tannery operations [21]. These pronounced differences underscore the strong influence of tannery activities on chromium contamination in surface waters. However, despite these alarming values, there is still a significant knowledge gap, as global data on chromium occurrence near tanneries remains scarce. Moreover, few studies provide comprehensive environmental assessments or risk analyses, leaving the true ecological and human health impacts of this contamination insufficiently understood.

In Portugal, the Alcanena region in the Santarém district is notable for its extensive tanning industry. This municipality hosts around 60 tannery companies, often associated with environmental and pollution concerns, particularly Cr release into the Alviela River. However, the practices of the 21st-century tanning industry are distinct from those of the 1980s and 1990s, which significantly shaped public perception [26]. To mitigate contamination, wastewater treatment plants (WWTPs) receiving effluents from this industry apply a specific treatment for Cr removal by means of its precipitation in the form of Cr hydroxide [27].

Therefore, the purpose of this study was to provide the first comprehensive evaluation of total Cr—whose oxidation states can readily interconvert—in surface waters impacted by effluents from the Alcanena tannery industry discharged into the Alviela River. Furthermore, we sought to assess the potential consequences of this contamination for both the aquatic environment and public health. This work offers novel insights by characterizing a pollution scenario that has remained largely underexplored to date.

## 2. Materials and Methods

### 2.1. Sampling Site and Collection

The WWTP that serves the tannery industry in this region discharges its effluents into the Carvalho Stream, a tributary of the Alviela River. The Carvalho Stream is a small, intermittent watercourse. It exhibits typical hydrological characteristics of Mediterranean-climate streams, with variable flow regimes influenced by seasonal precipitation patterns. During the wet season, it experiences increased discharge due to rainfall and runoff, while in the dry season, flow may be significantly reduced or even cease in certain stretches. The stream, as well as the Alviela River, interacts with local karstic systems, affecting groundwater recharge and surface water dynamics. The Alviela River flows for about 51 km before joining the Tagus River [28,29].

**Figure 1:**
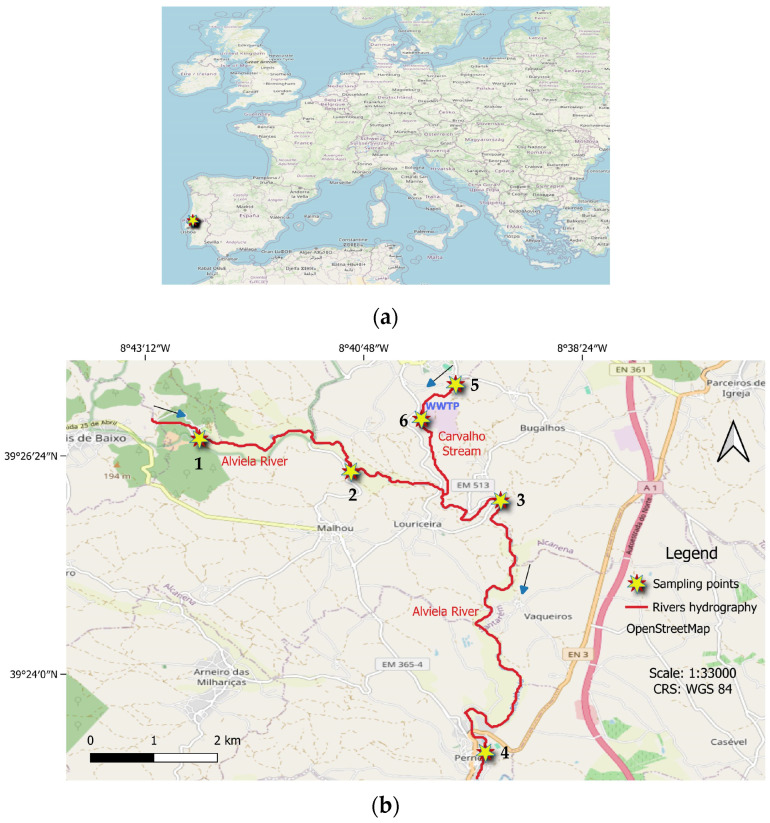
Sampling sites’ locations in Europe (**a**) and in Alcanena and Pernes municipalities (**b**).

Therefore, 53 grab samples of the dissolved phase of surface waters were collected from 6 different locations: 2 in the Carvalho Stream and 4 in the Alviela River, in the district of Santarém (Portugal) (Figure 1).

Sampling sites 1 to 4 were located in the Alviela River, with sites 1 and 2 located upstream of the Carvalho Stream and sites 3 and 4 situated downstream of the referred creek. Sampling locations 5 and 6 were in the Carvalho Stream, upstream and downstream, respectively, of the WWTP discharge. The sampling was carried out between February and June of 2021 and comprised 9 sampling campaigns (Table S2, Supporting Information). The river flow rate was also registered using a qualitative scale from 1 to 4 (Table S3, Supporting Information).

After collection into high-density polyethylene containers, previously rinsed with bi-distilled water, samples were refrigerated during transportation; on arrival to the lab, they were stored at −20 °C until analysis.

### 2.2. Sample Treatment and Analysis

Firstly, the water samples were tested for turbidity. When values were higher than 1 NTU (17 samples), the samples were microwaved (Table S4, Supporting Information). This was performed in a Millestone ETHOS touch control (Sorisole, Italy) with 0.5% nitric acid (nitric acid (68%) RS superpure from Carlo Erba (Milan, Italy)). When turbidity values were under 1 NTU, the analysis was performed without any sample treatment.

For samples with concentrations below 25 µg L^−1^, the analytical methodology used was graphite furnace atomic absorption spectrometry (GF-AAS) in an AAnalyst 800 from Perkin Elmer at 359.9 nm (Waltham, MA, USA) [30] (Table S4, Supporting Information).

Samples with a concentration above 25 µg L^−1^ were analyzed by ICP-OES in an Otima 2000DV from Perkin Elmer (Waltham, MA, USA) following the analytical methodology of ISO 11885:2007 at 267.716 nm [31] (Table S4, Supporting Information).

### 2.3. Validation and Quality Control

To ensure the quality control of these analytical methodologies, linearity was evaluated for both techniques.

Regarding the GF-AAS methodology, it was checked at 0, 1, 2, 3, and 4 µg L^−1^, presenting a detection and quantification limit of 0.33 and 1 µg L^−1^, respectively. The solution of the Cr ICP standard (1000 mg L^−1^), in the form of Cr(NO_3_)_3_, was purchased from Merk (Darmstadt, Germany). The reported uncertainty is an expanded uncertainty of 20% at a level of confidence of approximately 95% (using a coverage factor, k = 2, and assuming a normal distribution).

As for the ICP-OES methodology, the calibration curve was performed at 0, 25, 50, 100, and 250 µg L^−1^, and detection and quantification limits of 2.5 and 7.5 µg L^−1^, respectively, were achieved.

The expanded uncertainty is 27% (with a coverage factor, k = 2, for a confidence level of approximately 95%, assuming a normal distribution).

The obtained detection limits are similar to those obtained by other authors, which ranged between 0.04 and 1 µg L^−1^ [32,33,34].

Regarding quality control, to ensure that the analytical methodology delivered accurate results, in every analytical session, the limit of quantification was assessed using an independent standard. The calibration curve was verified every 10 samples with a control standard, accepted according to the corresponding control chart. In addition, every 20 samples, 1 sample was analyzed in duplicate, applying a maximum deviation criterion of 10%, and a recovery test was performed, with an acceptance range of 80–120%.

### 2.4. Statistical Analysis

Complete statistical analysis was performed using GraphPad Prism (8.4.3, GraphPad Software, Inc., San Diego, CA, USA). To test whether the datasets were of a Gaussian distribution, a D’Agostino–Pearson normality test was used. Since all of the datasets were not normally distributed, with non-homogeneous variances, nonparametric tests were applied. For the evaluation of three or more datasets, the Kruskal–Wallis test with Dunn’s post-test was used. For the comparison between two datasets, the Mann–Whitney test was performed. The statistical significance level was set to *p* < 0.05 [35]. When samples presented concentrations below the method quantification limit, half the method quantification limit was considered for statistical analysis. When samples’ concentrations were between the method detection limit and the method quantification limit, half the method quantification limit was used for the same purpose.

### 2.5. Environmental Risk Assessment

The evaluation of the environmental risk of the aquatic compartment was based on the determination of risk quotients. These were calculated by the ratio between measured environmental concentrations (minimum, mean, and maximum) and predicted no-effect concentrations on non-target organisms using three different trophic levels representative of the aquatic ecosystem (algae, daphnids, and fish) (Equation (1)). A potential environmental risk is indicated by a risk quotient higher than 1, whereas no risk is expected when the value is lower than 1 [36].

Equation (1)—risk quotient evaluation:

(1) $$Risk\;quotient=\frac{Measured\;environmental\;concentration}{Predicted\;no\text{-}effect\;concentration}$$

## 3. Results and Discussion

### 3.1. Frequency and Occurrence

Of the 53 samples analyzed, 38 (72%) exhibited Cr concentrations above the quantification limit, with an average of 71.4 µg L^−1^ and a maximum value of 560 µg L^−1^ (Table S4, Supporting Information).

**Figure 2:**
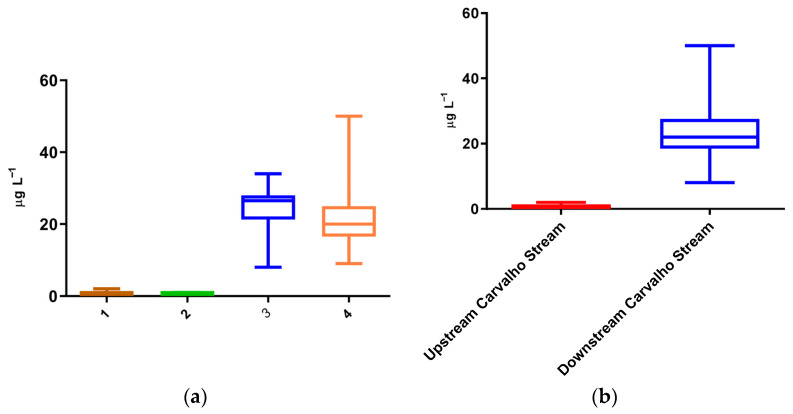
Occurrence of chromium in the Alviela River. All sampling points—1, 2, 3, and 4 (**a**) and comparison between upstream (1 and 2) and downstream (3 and 4) Carvalho Stream tributary (**b**).

In the Alviela River, sampling points 1 and 2, located upstream of the Carvalho Stream tributary, showed the lowest Cr concentrations in the study. At these sites, Cr levels remained below 2 µg L^−1^ on all samplings, with the majority of samples measuring under 1 µg L^−1^. No significant variations were observed at these points (Figure 2a).

At sampling points 3 and 4, located downstream of the Carvalho Stream tributary, Cr concentrations significantly increased (*p* < 0.014). At point 3, the average concentration was 24 µg L^−1^, ranging from 8 to 34 µg L^−1^. Similarly, point 4 showed an average of 22 µg L^−1^, with values ranging from 9 to 50 µg L^−1^, and no significant differences were observed between these two points. Despite the considerable distance between these two sites, of over 5 km, the recorded values remained comparable, suggesting that contamination persists downstream.

The disparity between upstream and downstream Cr concentrations in the Alviela River is evident in Figure 2b. Cr levels increased markedly after the confluence with the Carvalho Stream tributary, with average concentrations rising from 0.6 to 23 µg L^−1^, presenting significant differences (*p* < 0.0001) between sampling points 1 and 2 versus 3 and 4. Significant differences were also observed between individual upstream and downstream sampling points, confirming the impact of the Carvalho Stream, which receives WWTP discharge from the tannery industry, on the Alviela River.

**Figure 3:**
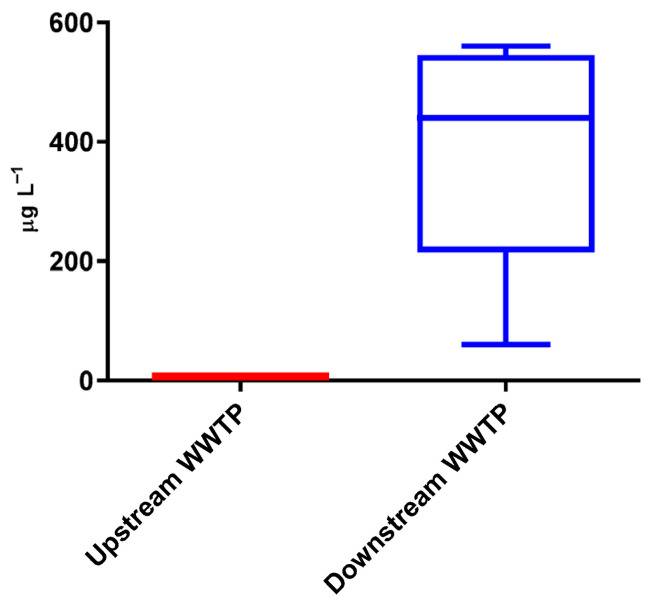
Occurrence of chromium in Carvalho Stream, upstream and downstream of the wastewater treatment plant.

In the Carvalho Stream, at sampling point 5 (upstream of the WWTP), the average Cr concentration was 5 µg L^−1^, ranging from 4 to 8 µg L^−1^ (Figure 3). In contrast, at point 6 (downstream of the WWTP), Cr concentrations were the highest among all sampling sites, with an average of 370 µg L^−1^ and values ranging from 60 to 560 µg L^−1^. These two points exhibited significant differences.

Given that the Cr levels at sampling point 6 were the highest among all collected samples, it can be inferred that the WWTP significantly contributes to the increase in Cr concentrations downstream, affecting both the Carvalho Stream and the Alviela River.

A comparison of Cr concentrations with the river flow rate in the Alviela River downstream of the Carvalho Stream revealed a correlation between these two variables; lower flow rates corresponded with higher Cr concentrations. This outcome was expected, as the contamination discharged from the WWTP is presumed to remain relatively constant each day, while the dilution factor varies. Furthermore, this finding raises concerns about the potential impact of climate change, particularly drought periods, on contaminant concentrations in water bodies [36]. Although no drought period occurred during the sampling period—which excluded the summer season—it is plausible that Cr concentrations during droughts could exceed those observed in this study. Additionally, the collection date and time had minimal influence on Cr concentration levels, showing no significant correlation.

When analyzing the limited number of publications on this subject, none of the Alcanena data from European and African countries show a large discrepancy (Table S1, Supporting Information). In Brazil and European countries, such as Poland, Cr average concentrations in rivers impacted by tanneries ranged from 0.7 to 19 µg L^−1^, lower than the values found in the current study (22–24 µg L^−1^) [22,24,25]. This supports the notion that the Alcanena region has a higher presence of Cr in the Alviela River, likely due to the tannery industry, compared to other European countries. On the other hand, African and Asian countries, such as Morocco, Algeria, and India, report much higher Cr concentrations in rivers affected by tanneries, ranging between 1170 and 5190 µg L^−1^. This likely reflects the use of older tanning methods in these countries, as well as ineffective or absent treatment of tannery effluents [21,23,24]. Therefore, our results are significantly higher compared to other developed countries, highlighting that mitigation strategies can be implemented to prevent this type of contamination.

### 3.2. Environmental and Human Impact

**Figure 4:**
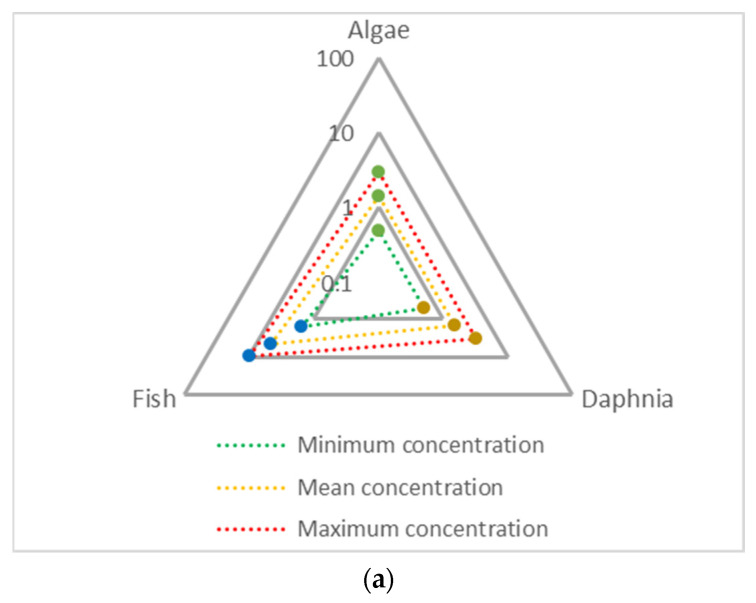

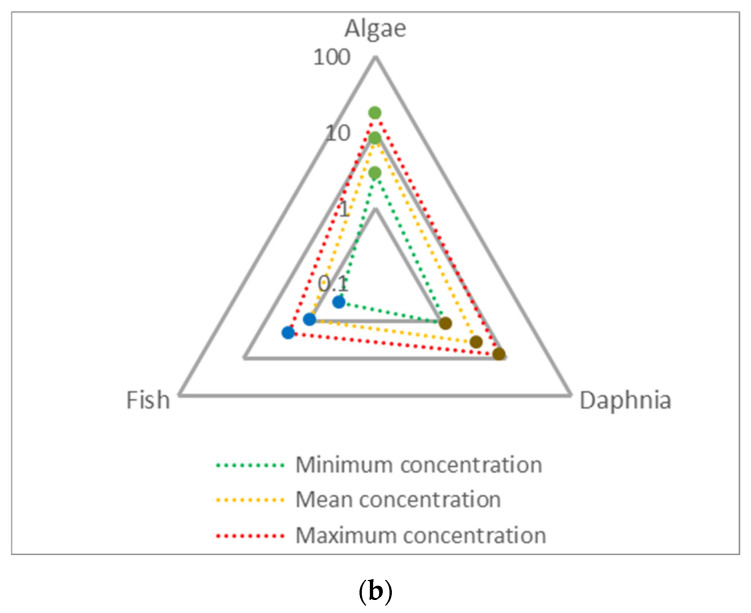
Risk quotients, using both acute (**a**) and chronic (**b**) toxicity data, in Alviela River samples downstream of the Carvalho Stream.

In the Alviela River samples collected upstream of the tributary, we can observe that no risk is expected. However, downstream of the tributary, all scenarios presented risk (Figure 4 and Table S5, Supporting Information) [14].

When observing the data for the maximum concentration, all trophic levels present risk with values up to 17.54. Using the mean concentration, only the risk quotient for chronic toxicity in fish (0.97) did not surpass 1, with values up to 8.15 for chronic toxicity in algae. Even when using the minimum concentration found in the Alviela River, from samples collected downstream of the Carvalho Stream, risk is presented for fish (1.54) and algae (2.81) for both acute and chronic toxicity, respectively. Additionally, the concentrations found also surpassed various EQSs from different countries (acute toxicity: 16 µg L^−1^; chronic toxicity: 1–11 µg L^−1^) [14].

The samples in the Carvalho Stream, downstream of the WWTP discharge, presented risk quotients of up to 21.1, 129.3, and 196.5 for the minimum, mean, and maximum concentrations detected, respectively. This highlights the impact of WWTP effluent on the receiving body of water.

This risk assessment highlights the potential for Cr toxicity in the aquatic environment. The concentrations detected may affect multiple trophic levels, including algae, invertebrates, and vertebrates (notably fish), and thereby disrupt ecosystem function [5,14]. Moreover, it has also been reported that even at environmentally relevant concentrations, Cr toxicity can increase in the presence of microplastics, which, although not assessed in this study, are ubiquitous in the aquatic systems. This additional factor may further influence the observed results [37].

Furthermore, 41% of the samples in the Alviela River (downstream of the Carvalho Stream) surpassed the proposed Cr concentration for drinking water in Portugal (25 µg L^−1^), posing a potential risk to the region’s water supply sustainability. In the Malhou village, located near the most contaminated sampling points in the Alviela River, Cr concentrations in drinking water reached 20 µg L^−1^, probably due to the impact of river water. In fact, at this location, water treatment consists only of chlorination, not mitigating the potential risk from this contamination but increasing it, since chlorination promotes oxidation of Cr (III) to Cr (VI) [38]. Therefore, this contamination can impact water supply sustainability due to the possible need for additional treatments or the adoption of different water sources. These values in Malhou village contrast with nearby villages in the Alcanena council area, such as Alcanena, Carvalheiro, Espinheiro, and Minde, which are not impacted by the Alviela River and showed Cr levels below the water management company’s detection limit (6 µg L^−1^) [16,39].

In addition to the direct risks posed by the presence of Cr in watercourses, the river water is commonly used for agricultural irrigation in the Alviela basin. This practice can disseminate contamination to crops and to animal feed produced in the area, potentially extending Cr toxicity beyond the aquatic environment and into the food chain [5,40]. In line with the One Health concept, these findings raise concerns that Cr contamination may not be confined to the environment. Its potential presence in the food chain and drinking water could pose health risks to the local population.

## 4. Conclusions

This study confirms that average Cr concentrations in the Alviela River increase significantly (0.6 to 23 µg L^−1^) downstream of the Carvalho Stream, where the WWTP discharges effluent from the tannery industry. The average concentration of Cr (22 µg L^−1^) remained high several kilometers downstream, highlighting its long-term environmental impact. These findings show that Cr levels in the Alviela River are higher than those reported in other developed countries (0.7–19 µg L^−1^), but lower than those reported in some African and Asian countries (1170–5190 µg L^−1^). Nonetheless, the recorded values exceed EQSs (1–16 µg L^−1^) and predicted no-effect concentrations (2.9–23.9 µg L^−1^), posing a risk to aquatic ecosystems. Risk quotients exceeded 1 for all trophic levels, reaching values of up to 8.15 when using mean Cr concentrations measured in the Alviela River, downstream of the Carvalho Stream tributary. River water used for irrigation may spread contamination to crops, while nearby villages like Malhou showed higher Cr in drinking water, raising health concerns.

These findings reinforce the need for better management of tannery effluents, stricter regulations, and continuous monitoring, namely of sediments and food. Addressing these issues aligns with the One Health concept, as environmental contamination can have direct consequences on public health.

## Data Availability

Data is contained within the article or Supplementary Material. Further inquiries can be directed to the corresponding author.

## Supplementary Materials

The following supporting information can be downloaded at https://www.mdpi.com/article/10.3390/toxics13121068/s1: Table S1: Average chromium concentrations in rivers impacted by tannery industry; Table S2: Sampling dates and hours in the different locations; Table S3: Flow rate in the Alviela River; Table S4: Chromium concentration at the different sampling points; Table S5: Risk quotients in Alviela River samples downstream of the Carvalho Stream.

## Abbreviations

The following abbreviations are used in this manuscript:

Cr	Chromium
EQS	Environmental quality standards
ICP-OES	Inductively coupled plasma coupled with optical emission spectrometry detection
ICP-MS	Inductively coupled plasma coupled with spectrometry detection
WWTPs	Wastewater treatment plants
