# Supplementary material for: Assessment of Chromium Contamination in Aquatic Environments near Tannery Industries: A Portuguese Case Study
Source: Toxics. 2025 Dec 11;13(12):1068. doi: 10.3390/toxics13121068 (PMC12737648; doi:10.3390/toxics13121068)
Supplement: Supplementary file 1 [file toxics-13-01068-s001.zip › toxics-3987393-supplementary.pdf]

Assessment of Chromium Contamination in Aquatic Environments near Tannery Industries: A Portuguese Case Study

Liliana J. G. Silva, Maria J. G. Casimiro, Angelina Pena, Maria J. Campos and André M. P. T. Pereira *

LAQV, Requimte, Laboratory of Bromatology and Pharmacognosy, Faculty of Pharmacy, University of Coimbra, Polo III, Azinhaga de Stª Comba, 3000-548 Coimbra, Portugal; ljgsilva@ff.uc.pt (L.J.G.S.); mariacasimiro15@gmail.com (M.J.G.C.); apena@ci.uc.pt (A.P.); mcampos@ff.uc.pt (M.J.C.)

* Correspondence: andrepereira@ff.uc.pt

Table S1. Average chromium concentrations in rivers impacted by tannery industry.

Country	River	Concentration ($\mu g\ L^{-1}$)	Sampling year	Ref.
**Poland**	Dunajec	19	2001-2004	[1]
**Poland**	Wieprz	8	2009	[2]
**Morocco**	Sebou	1400	2002	[1]
**Algeria**	Mouttas	1170-1463	2007	[3]
**Brasil**	Candeias/Graças/Preto	0.65-8.6	2013-2014	[4]
**India**	Hazaribagh area	5190	-	[5]

Table S2. Sampling dates and hours in the different locations.

**Sampling date**	**Sampling location and collection hour**					
	1	2	3	4	5	6
**23/2/2021**	12h00	13h12	12h39	12h56	12h20	12h28
**3/3/2021**	14h13	13h53	-	15h21	14h45	14h54
**11/3/2021**	10h00	09h45	10h40	10h24	10h15	10h55
**20/3/2021**	10h46	10h30	11h46	12h02	11h25	11h34
**26/3/2021**	14h15	15h50	15h50	16h04	16h30	16h37
**1/4/2021**	9h47	10h00	9h07	8h45	9h33	9h23
**9/4/2021**	9h37	9h27	10h19	10h38	9h54	10h06
**19/4/2021**	16h35	16h47	15h53	15h33	15h33	16h05
**20/6/2021**	14h35	14h25	15h35	15h45	15h15	15h25

Table S3. Flow rate in the Alviela River.

Sampling date	Flow rate
23/02/2021	4
3/03/2021	3
11/03/2021	3
20/03/2021	2
26/03/2021	2
1/04/2021	2
9/04/2021	2
19/04/2021	2
20/06/2021	1

1—water was still in the small dam in the river; 2—the water height was below the top of the small dam in the river; 3—the water height was near the top of the small dam in the river; 4—the water was over the height of the small dam in the river.

Table S4. Chromium concentration at the different sampling points.

	1	2	3	4	5	6
23/02/2021	<LOD<br>a, d	<LOD<br>a, c	8 µg/L<br>a, c	9 µg/L<br>a, c	8 µg/L<br>a, c	60 µg/L<br>b, d
3/03/2021	<LOD<br>a, c	<LOD<br>a, d	-	15 µg/L<br>a, c	7 µg/L<br>a, c	180 µg/L<br>b, d
11/03/2021	<LOD<br>a, d	<LOD<br>a, d	22 µg/L<br>a, c	18 µg/L<br>a, c	5 µg/L<br>a, c	290 µg/L<br>b, d
20/03/2021	<LOD<br>a, d	<LOD<br>a, d	28 µg/L<br>b, d	26 µg/L<br>b, d	4 µg/L<br>a, c	560 µg/L<br>b, d
26/03/2021	<LOD<br>a, d	<LOD<br>a, d	34 µg/L<br>b, d	24 µg/L<br>a, c	4 µg/L<br>a, c	530 µg/L<br>b, d
1/04/2021	<LOD<br>a, d	<LOD<br>a, d	26 µg/L<br>b, d	20 µg/L<br>a, c	4 µg/L<br>a, c	440 µg/L<br>b, d
9/04/2021	1 µg/L<br>a, c	<LOD<br>a, d	27 µg/L<br>b, d	20 µg/L<br>a, c	4 µg/L<br>a, d	460 µg/L<br>b, d
19/04/2021	2 µg/L<br>a, d	1 µg/L<br>a, d	21 µg/L<br>a, d	19 µg/L<br>a, d	4 µg/L<br>a, d	250 µg/L<br>b, d
20/06/2021	<LOD<br>a, d	<LOD<br>a, d	28 µg/L<br>b, d	50 µg/L<br>b, d	6 µg/L<br>a, d	560 µg/L<br>b, d

a—graphite furnace atomic absorption spectrometry (GF-AAS); b—inductively coupled plasma–optical emission spectrometry (ICP-OES); c—samples microwaved; d—samples not microwaved

Table S5. Risk quotients in Alviela River samples downstream of the Carvalho Stream.

		Acute toxicity			Chronic toxicity		
		Algae	Invertebrates	Fish	Algae	Invertebrates	Fish
PNECs (μg L^{-1})		16.76	15.56	5.208	2.85	6.39	23.88
Minimum concentration	8 μg L^{-1}	0.48	0.51	1.54	2.81	1.25	0.34
Mean concentration	23.24 μg L^{-1}	1.39	1.49	4.46	8.15	3.64	0.97
Maximum concentration	50 μg L^{-1}	2.98	3.21	9.60	17.54	7.82	2.09

PNEC: predicted no-effect concentration [6].

References

1. Dominik, J.; Vignati, D.A.L.; Koukal, B.; Pereira de Abreu, M.H.; Kottelat, R.; Szalinska, E.; Baś, B.; Bobrowski, A. Speciation and environmental fate of chromium in rivers contaminated with tannery effluents. *Eng. Life Sci.* **2007**, *7*, 155–169, doi:10.1002/elsc.200620182.

2. Wolińska, A.; Stępniewska, Z.; Włosek, R. The influence of old leather tannery district on chromium contamination of soils, water and plants. *Nat. Sci.* **2013**, *05*, 253–258, doi:10.4236/ns.2013.52A037.

3. Leghouchi, E.; Laib, E.; Guerbet, M. Evaluation of chromium contamination in water, sediment and vegetation caused by the tannery of Jijel (Algeria): a case study. *Environ. Monit. Assess.* **2009**, *153*, 111–117, doi:10.1007/s10661-008-0341-3.

4. de Sousa, E.A.; Luz, C.C.; de Carvalho, D.P.; Dorea, C.C.; de Holanda, I.B.B.; Manzatto, Â.G.; Bastos, W.R. Chromium distribution in an Amazonian river exposed to tannery effluent. *Environ. Sci. Pollut. Res.* **2016**, *23*, 22019–22026, doi:10.1007/s11356-016-7443-1.

5. ANAWAR, H.M.; SAFIULLAH, S.; YOSHIOKA, T. Environmental Exposure Assessment of Chromium and Other Tannery Pollutants at Hazaribagh Area, Dhaka, Bangladesh, and Health Risk. *J. Environ. Chem.* **2000**, *10*, 549–556, doi:10.5985/jec.10.549.

6. Alves Miranda, G.; Soares dos Santos, F.; Lourenço Pereira Cardoso, M.; Etterson, M.; C. Amorim, C.; V. M. Starling, M.C. Proposal of novel Predicted No Effect Concentrations (PNEC) for metals in freshwater using Species Sensitivity Distribution for different taxonomic groups. *Sci. Rep.* **2025**, *15*, 8180, doi:10.1038/s41598-025-92692-4.
